# Targeting the Spinal Cord‐Brain Axis: Electroacupuncture Mitigates Remote Frontal Cortex Neuroinflammation via HMGB1/TLR4 to Aid Functional Recovery After Spinal Cord Injury

**DOI:** 10.1002/brb3.71215

**Published:** 2026-01-19

**Authors:** Yu Ning, Xin Hao, Phattharapon Rattanasakon, Yifei Dong, Ying Yang, Keduo Liu, Yuting Lin, Suhua Shi, Yuping Mo, Zhigang Li

**Affiliations:** ^1^ School of Acupuncture, Moxibustion and Tuina Beijing University of Chinese Medicine Beijing China; ^2^ Department of Rehabilitation The Third Affiliated Hospital of Beijing University of Chinese Medicine Beijing China; ^3^ College of Clinical Medicine Hainan Vocational College of Medicine Haikou China; ^4^ Traditional Chinese Medicine Department Shenzhen Third People's Hospital Southern University of Science and Technology Shenzhen China

**Keywords:** electroacupuncture, HMGB1/TLR4 /NF‐κB pathway, microglial activation, motor neuron survival, neuroinflammation, spinal cord injury

## Abstract

**Background:**

Beyond the primary lesion, spinal cord injury (SCI) induces secondary neuroinflammation in the frontal cortex, a critical factor exacerbating neural damage and impeding functional recovery. This remote inflammatory response is predominantly regulated by the HMGB1/TLR4 signaling pathway. While electroacupuncture (EA) shows therapeutic promise, whether its efficacy is causally dependent on modulating this supraspinal inflammation remains unproven. This study investigated whether EA promotes functional recovery by suppressing the HMGB1/TLR4/NF‐κB pathway in the frontal cortex of rats with SCI.

**Methods:**

One hundred and fifty adult male rats were randomly assigned to Sham, SCI, EA, SCI+ HMGB1 Inhibitor (I), and SCI + EA + HMGB1 Inhibitor (EA + I) groups. Functional recovery was assessed at 1, 7, 14, and 28 days post‐SCI using Basso, Beattie, and Bresnahan (BBB) scores and the inclined plate test. Frontal cortex tissue was analyzed at 7, 14, and 28 days post‐injury for proteins in the HMGB1/TLR4/NF‐κB pathway, TNF‐α (via Western blot and immunofluorescence), and microglial activation (Iba‐1 via immunofluorescence). The mRNA levels of these targets were assessed by qPCR at 7 and 28 days. Spinal cord tissue was evaluated for axonal integrity (NF200 via immunofluorescence) and for motor neuron survival (Nissl staining) at 28 days post‐injury.

**Results:**

EA treatment significantly improved locomotor and postural recovery compared to the SCI group. Concurrently, EA suppressed the SCI‐induced upregulation of HMGB1, TLR4, NF‐κB, and TNF‐α in the brain throughout the subacute (Day 7), transition (Day 14), and chronic (Day 28) phases, thereby inhibiting microglial activation. This neuroprotective effect was accompanied by spinal motor neuron survival and axonal integrity. The co‐administration of an HMGB1 inhibitor with EA established the pathway's necessity by showing that the resulting potentiation of therapeutic outcomes positions the HMGB1/TLR4/NF‐κB axis as a central mechanism for EA.

**Conclusion:**

EA effectively ameliorates motor dysfunction following SCI by attenuating neuroinflammation in the frontal cortex. The underlying mechanism is causally linked to the downregulation of the HMGB1/TLR4/NF‐κB signaling axis. The enhanced neuroprotection observed when combining EA with HMGB1 inhibition validates this signaling axis as the primary and essential target for EA's therapeutic effects in SCI management.

## Graphical abstract

Spinal cord injury (SCI) has been shown to trigger remote neuroinflammation in the frontal cortex via the HMGB1/TLR4/NF‐κB pathway, exacerbating neural damage and impairing functional recovery. The present study demonstrates that electroacupuncture (EA) at GV4 (Mingmen) and GV14 (Dazhui) effectively mitigates this pathological cascade. EA treatment significantly suppressed HMGB1/TLR4/NF‐κB activation and TNF‐α expression in the frontal cortex across the acute, subacute, and chronic phases post‐SCI, accompanied by reduced microglial activation (Iba‐1+ cells). Mechanistically, EA promoted the preservation of spinal motor neurons and axonal integrity (NF200+ fibers), which was associated with an enhancement in locomotor function (Basso, Beattie, and Bresnahan (BBB) scores, inclined plate test). The combination of treatment with an HMGB1 inhibitor (glycyrrhizin) and EA resulted in a potentiation of the therapeutic effects of EA, thereby confirming the HMGB1/TLR4 axis as a critical target for EA's neuroprotective actions. These findings underscore the potential of EA as a promising strategy to modulate the spinal cord‐brain inflammatory axis and enhance functional recovery after SCI.

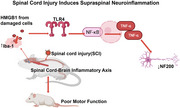

## 1 Introduction

Spinal Cord Injury (SCI) is a serious neurological condition, typically leading to significant physical impairment, psychological stress, and  economic burden(Karsy and Hawryluk 2019). Neuroinflammation is increasingly recognized as a critical determinant negatively impacting the overall recovery trajectory post‐SCI (Hellenbrand et al. 2021). Despite comprehensive studies focusing on inflammatory processes in the damaged spinal cord regions (Freyermuth‐Trujillo et al. 2022; Sunshine et al. 2023), accumulating evidence reveals that SCI also elicits region‐specific neuroinflammatory responses in the frontal cortex (Li et al. 2020; Li, Ritzel, et al. 2020a). These supraspinal neuroinflammatory sequelae have profound implications for structural and functional brain remodeling, thereby highlighting an urgent need for integrated therapeutic strategies that address both local and remote pathologies.

Within the complex inflammatory cascade following SCI, high mobility group box 1 (HMGB1) protein acts as a pivotal alarmin. Released acutely post‐injury, HMGB1 contributes to the compromise of blood‐spinal cord and blood‐brain barrier integrity and perpetuates neuroinflammation (Freitas‐Andrade et al. 2023; Scaffidi et al. 2002). Specifically, HMGB1 interacts with TLR4, a receptor highly expressed on microglial cells (Ohnishi et al. 2014). TLR4 activation subsequently triggers a downstream inflammatory response, enhancing pro‐inflammatory cytokine release and intensifying secondary injury, including in remote brain regions. Consequently, the HMGB1‐TLR4 signaling axis is recognized as a crucial mechanistic link between the primary spinal lesion and the ensuing neuroinflammatory responses in the brain. The intricate interplay involving HMGB1, TLR4, and the resultant neuroinflammatory cascade thus presents promising therapeutic targets (Sun et al. 2017).

Accumulating evidence demonstrates that SCI instigates widespread neuroinflammatory changes extending to various brain regions (Wu et al. 2024). It is hypothesized that inflammatory mediators, including HMGB1, released from the injured spinal cord, can propagate their effects to distant brain regions via the TLR4 signaling axis. This remote neuroinflammation contributes to disruptions to neural connectivity, altered neurotransmitter dynamics, and subsequent impairments in cognitive and motor functions. Therefore, therapeutic strategies targeting this disseminated inflammatory response could not only foster spinal repair but also ameliorate secondary brain dysfunction, ultimately enhancing overall functional recovery.

Acupuncture, a therapeutic modality with ancient origins, has long been employed for neurological disorders and neurological injury (Li et al. 2023; Vickers et al. 2018; Yu et al. 2021). Modern research on electroacupuncture (EA) has substantiated its therapeutic efficacy. Randomized controlled trials and preclinical studies have shown that EA significantly promotes motor recovery following central nervous system (CNS) trauma, such as ischemic stroke and spinal trauma (Lin et al. 2017; Yang et al. 2015). Collectively, these findings underscore EA as a promising adjunctive therapy capable of exerting multimodal neuroprotective effects to optimize functional outcomes in SCI. Among various acupoints, those on the Governor Vessel (Du Mai), which runs along the spinal column, are considered particularly crucial for treating spinal and neurological disorders (Li et al. 2022; Xu et al. 2021).

Our selection of GV4 (Mingmen) and GV14 (Dazhui) is grounded in their well‐established roles in both traditional theory and modern research. GV14, located below the C7 spinous process, is a major confluence point of Yang meridians and anatomically proximal to crucial spinal segments. Studies have demonstrated that EA at GV14 suppresses neuroinflammation, potentially by inhibiting microglial activation and downregulating pro‐inflammatory cytokines (Hou et al. 2025; Liu et al. 2020). GV4, known as the “Gate of Life,” located on the lumbar region, is traditionally believed to nourish the spinal cord and strengthen constitutional function. Modern research corroborates that EA at GV4 promotes motor neuron survival and enhances functional restoration in SCI scenarios (Wang et al. 2021; Zhao et al. 2023). Furthermore, both acupoints are located on the Governor Vessel, which is intrinsically linked to the CNS, making them ideal candidates for modulating both local spinal pathology and remote supraspinal responses. Building upon this potential to influence remote supraspinal responses, the specific impact of EA at GV4 and GV14 on SCI‐induced neuroinflammation in distant brain regions and the molecular underpinnings, particularly the engagement of the HMGB1/TLR4 pathway, are yet to be thoroughly investigated.

Building upon this, we hypothesized that EA at Governor Vessel acupoints would enhance functional recovery after SCI by attenuating neuroinflammation within the frontal cortex. We proposed the underlying mechanism involves the suppression of the HMGB1/TLR4/NF‐κB signaling cascade.

Our primary outcome was locomotor function, assessed by Basso, Beattie, and Bresnahan (BBB) scores and the inclined plate test at 1, 7, 14, and 28 days post‐injury. Secondary outcomes included molecular markers of the HMGB1/TLR4/NF‐κB pathway and microglial activation (Iba‐1) in the frontal cortex, as well as spinal cord axonal integrity (NF200) and motor neuron survival (Nissl staining), evaluated at 7 and 28 days post‐injury.

## 2 Materials and Methods

### 2.1 Animals

Specific Pathogen‐Free (SPF) male Sprague‐Dawley (SD) rats (220±20 g, 6–7 weeks old) were sourced from Sibeifu (Beijing) Biology Technology Co., Ltd [Animal Lot: SCXK (Jing) 2019‐0010]. The animals were kept in SPF conditions at the Beijing University of Chinese Medicine's Animal Experimental Center. The conditions were meticulously controlled, featuring temperatures between 23°C and 24°C, humidity ranging from 45% to 60%, and a consistent 12‐h light and dark schedule. Food and water were available ad libitum. Every experiment involving animals was rigorously reviewed and cleared by the Animal Experimental Center of Beijing University of Chinese Medicine (BUCM2024051002‐2081) and strictly followed the “Guiding Opinions on the Treatment of Experimental Animals” from the Ministry of Science and Technology of the PRC. The research adhered to the ARRIVE (Animal Research: Reporting of In Vivo Experiments) guidelines throughout its execution and reporting.

### 2.2 Experimental Design and Animal Allocation

Following a three‐day acclimatization period, a total of 150 SD rats were used. The sample size was determined using a pre‐study power analysis (G*Power 3.1) to identify a substantial effect size (*f* = 0.8), ensuring 80% power with a significance level of 0.05 for the primary behavioral outcomes. The animals were randomly divided into five groups (*n* = 10 per group): Sham‐operation Group (Sham), SCI model Group (SCI), Electroacupuncture + SCI Group (EA), HMGB1 Inhibitor + SCI Group (I), and HMGB1 Inhibitor + EA + SCI Group (EA + I). To facilitate both longitudinal functional assessment and endpoint‐based molecular analysis, the rats within each group were further assigned to three terminal endpoint cohorts: a 7‐day cohort (n = 10 per group), a 14‐day cohort (*n* = 10 per group), and a 28‐day cohort (*n* = 10 per group).

All animals underwent behavioral testing on post‐injury days 1, 7, 14, and 28. After the day 7 test, rats in the 7‐day cohort were euthanized for tissue collection. Subsequent to the completion of the 14‐day test, rats within the 14‐day cohort were euthanized for the purpose of tissue collection. Rats in the 28‐day cohort continued to receive respective treatment and underwent further behavioral testing on days 14 and 28, after which they were euthanized for tissue collection.

#### 2.2.1 Behavioral Assessment

All animals underwent behavioral testing at each designated time point prior to any scheduled euthanasia. Consequently, the sample sizes for the longitudinal behavioral data were as follows: ***n* = 30** per group on day 1 and day 7 (including animals from the 7‐, 14‐, and 28‐day cohorts); ***n* = 20** per group on day 14 (including animals from the 14‐ and 28‐day cohorts); and ***n* = 10** per group on day 28 (animals from the 28‐day cohort only).

#### 2.2.2 Molecular and Histological Analyses

Tissues were collected exclusively at the designated terminal endpoints. At each endpoint (day 7, 14, and 28), *n* = 10 rats per group were euthanized. To accommodate conflicting tissue processing requirements, the animals within each endpoint cohort were randomly allocated into two subgroups: **5 rats** were designated for molecular analyses (requiring fresh frozen tissue), and the **remaining 5 rats** were designated for histological assessment (requiring perfusion fixation). From each animal, both the frontal cortex and the injured spinal cord segment were harvested to ensure paired data for supraspinal and spinal analyses. This design resulted in *n* = 5 independent biological replicates per assay, per group, for each time point.

### 2.3 Surgical Procedure

Rats were anesthetized with isoflurane (2.5% for induction, 1.5–2.0% for maintenance) and placed in a prone position. Under aseptic conditions, a dorsal midline incision was made, and a T10 laminectomy was performed, with meticulous attention to preserving the integrity of the dura mater. For the SCI group, a moderate contusion injury was induced using a modified Allen's weight‐drop device by dropping a 10‐gram weight from a 40‐millimeter height (40 g·cm) onto the exposed spinal cord. The efficacy of the injury induction procedure was substantiated by the observation of intraoperative hematoma and subsequent bilateral hindlimb paralysis.

After the impact, a sterile gelatin sponge was placed over the laminectomy site to ensure hemostasis. Subsequently, the muscle and skin were sutured in layers using a 4‐0 suture. Immediately following the surgical procedure, each rat was administered an intramuscular injection of penicillin (40,000 units) to forestall any potential infections. Animals in the Sham group underwent the same T10 laminectomy but did not receive the spinal cord impact.

### 2.4 Postoperative Care

Immediately after surgery, animals were placed on a heating pad to maintain body temperature until they had fully recovered from the effects of anesthesia. To alleviate postoperative pain, all animals received ibuprofen (approximately 30 mg/kg/day, based on average water consumption) administered in their drinking water for the first 3 days post‐surgery. Manual bladder expression was performed twice daily until spontaneous urination was restored. All animals were housed with free access to food and water, and their general health status was monitored daily.

All procedures were approved by the Animal Ethics Committee of Beijing University of Chinese Medicine (BUCM2024051002‐2081) and were conducted in strict accordance with the National Institutes of Health Guide for the Care and Use of Laboratory Animals.

### 2.5 HMGB1 Inhibitor Administration

Glycyrrhizin (PHL89217, Sigma‐Aldrich, USA), a specific inhibitor of HMGB1, was dissolved in sterile saline. Rats in the I and EA + I groups received an intraperitoneal (i.p.) injection of glycyrrhizin at a dose of 20 mg/kg. The first injection was administered 30 min after the induction of SCI, followed by once‐daily injections until the experimental endpoints, day 14 and day 28. This dosage and administration protocol was based on previous studies demonstrating its efficacy in CNS injury models (Lou et al. 2025). To control for the potential effects of the injection procedure itself, rats in the Sham, SCI, and EA groups received i.p. injections of an equivalent volume of sterile saline (vehicle) on the same schedule.

### 2.6 Electroacupuncture Treatment

EA treatment commenced on the second day post‐surgery and was administered once daily for 20 min per session, continuing until the experimental endpoint (day 28). The treatment was administered to the EA and EA + I groups. For the procedure, Dazhui (GV14) was located in the depression below the C7 spinous process, and Mingmen (GV4) was located below the L2 spinous process. At GV14, a disposable sterile needle (0.30 × 25 mm; Zhongyan Taihe) was inserted obliquely downward; at GV4, a second needle was inserted obliquely upward, both to a depth of 5–10 mm. The needles were connected to a HANS‐LH202 stimulator, with the cathode at GV14 and the anode at GV4. A disperse‐dense wave (2/100 Hz, 1 mA) was applied, with the intensity adjusted to induce mild, non‐distressing muscle twitching.

Rats in the Sham, SCI, and I groups did not receive any active postoperative therapy. To control for the potential confounding effects of handling and restraint, rats in these groups underwent a sham handling procedure. This involved gentle grasping and restraint for 20 min, performed concurrently with each electroacupuncture session administered to the EA group. This procedure ensured that all animals experienced comparable levels of handling and exposure to the experimental environment, thereby minimizing stress‐related confounding variables.

### 2.7 Basso, Beattie, and Bresnahan (BBB) Locomotor Rating Scale

Hindlimb functional recovery was evaluated using the BBB locomotor rating scale (0 = complete paralysis; 21 = normal locomotion) Basso 1995. On post‐surgery days 1, 7, 14, and 28, each rat's performance was observed for at least 3 min in an open field by two pre‐trained investigators, both unaware of the group assignments. The average of their scores was used to determine the final BBB score.

### 2.8 Inclined Plate Test

Motor function evaluations were conducted via the inclined plate test on post‐surgery days 1, 7, 14, and 28 Rivlin 1977. The inclined plate test was conducted at least 2 h after the BBB test on the same day to allow for a sufficient rest period and minimize animal fatigue. The apparatus consisted of an inclinable wooden board equipped with a calibrated electronic goniometer. For each trial, rats were placed on the board facing the upward slope of the incline, with their body axis perpendicular to the platform's tilt axis. The board was then gradually tilted at approximately 5° every 10 s. The uppermost angle allowing the rat to hold its stance for no less than 5 s was documented. Each rat underwent three trials, with an inter‐trial rest period of 2 min. The average angle from the three trials constituted the maximum maintainable angle. Greater angles indicate superior motor function. The assessment was performed by a researcher unaware of the group allocations.

### 2.9 Euthanasia and Tissue Collection

At the designated endpoints (7, 14, and 28 days post‐injury), following the final behavioral assessments, rats were euthanized for tissue collection. Rats received a lethal dose of sodium pentobarbital (100 mg/kg, i.p.), with respiratory and cardiac arrest confirming mortality. For the histological subgroup (*n* = 5), rats were transcardially perfused with ice‐cold saline followed by 4% paraformaldehyde (PFA). Following perfusion, the frontal cortex and a 20‐mm section of the spinal cord centered around the T10 lesion were carefully removed. These tissues were then post‐fixed in 4% PFA and processed for paraffin embedding. For the molecular subgroup (*n* = 5), rats were euthanized by anesthetic overdose. The frontal cortex and the spinal cord tissues were rapidly dissected, snap‐frozen in liquid nitrogen, and stored at −80°C for subsequent Western blot and qPCR analyses.

### 2.10 Immunofluorescence Assay

Sections (4 µm, paraffin‐embedded) underwent xylene deparaffinization and subsequent rehydration via graded ethanol. Citrate buffer (pH 6.0) heated sections for antigen unmasking (water bath, 75–85°C, 10 min), then PBS washes (pH 7.4, 3 × 5 min). Sections were then outlined with a hydrophobic barrier pen and blocked with 3% BSA in PBS for 30 min at room temperature. Overnight, sections were then incubated at 4°C with primary antibodies in PBS containing 1% BSA, at the dilutions listed below: Iba1 antibody (ab178846; Abcam; 1:200), HMGB1 antibody (ab18256; Abcam; 1:200), NF‐κB p65 antibody (ab32536; Abcam; 1:250), TLR4 antibody (ab22048; Abcam; 1:200), TNF‐α antibody (ab183218; Abcam; 1:200), and NF (Neurofilament H) antibody (ab215903; Abcam; 1:200). After three 5‐minute PBS washes, sections were incubated with Alexa Fluor 488 Donkey Anti‐Rabbit IgG secondary antibody (1:500; ab150073, Abcam) for 1 h at room temperature in the dark. Following the primary antibody incubation targeting neurofilament H (mouse host), sections were washed thoroughly. Subsequently, they were incubated with Alexa Fluor 594 Donkey Anti‐Mouse IgG (1:500; ab150108, Abcam) for an hour at room temperature, shielded from light. Sections were counterstained with DAPI to visualize nuclei; this was followed by rinsing thrice with PBS. Finally, sections were mounted with an antifade medium and imaged using a fluorescence microscope (Olympus BX43) at 400× magnification. For quantification, three non‐overlapping fields of view were randomly imaged per section from three separate sections per animal. The data from all fields and sections for a single animal were averaged to generate one biological replicate value (*n* = 1). Therefore, the animal served as the statistical unit for all analyses. ImageJ/Fiji software was used for all quantifications by an investigator blinded to the experimental groups.

### 2.11 Western Blot Assay

Frontal cortex samples were disrupted with chilled RIPA buffer (Servicebio, China) for the extraction of total proteins. Protein levels were quantified via BCA assay (Servicebio, China). Protein samples, each containing the same amount of protein (20 µg), were run on 10% SDS‐PAGE gels to separate them by size. After that, we transferred the separated proteins onto PVDF membranes (Millipore, USA) using a semi‐dry transfer device. Samples were incubated with 5% skim milk in TBST buffer for an hour at ambient temperature. Next, membranes were exposed to primary antibodies (Servicebio, China) in TBST at 4°C overnight: anti‐HMGB1 (1:1000; Rabbit; GB11103‐100), anti‐TLR4 (1:1000; Rabbit; GB11519‐100), anti‐NF‐κB p65 (1:1000; Rabbit; GB11997‐100), and anti‐TNF‐α (1:1000; Rabbit; GB115726). *β*‐actin (1:5000; Rabbit; GB15003, Servicebio, China) was used as an internal loading control. Following three 10‐min washes with TBST, membranes were incubated with a horseradish peroxidase (HRP)‐conjugated goat anti‐rabbit secondary antibody (1:5000; GB23303, Servicebio, China) for 1.5 h at room temperature. Protein bands were identified with Servicebio's ECL reagent G2161‐200ML and analyzed via a Bio‐Rad ChemiDoc MP imaging system. Band densities were quantified using ImageJ software. Each band's intensity was normalized to its corresponding β‐actin loading control. Each lane on the gel represented a sample from an individual animal. The animal, therefore, served as the independent biological replicate (*n* = 1) and the statistical unit for all subsequent analyses.

### 2.12 Quantitative Real‐time‐PCR (qPCR)

RNA was isolated from the frontal cortex via RNAex Pro Reagent (AG21102, Accurate Biotechnology, Hunan, China), adhering to the provided protocol. Complementary DNA (cDNA) was synthesized using the Evo M‐MLV RT Mix Kit (AG11728, Accurate Biotechnology), and qPCR was executed with the SYBR Green Premix Pro Taq HS qPCR Kit (AG11701, Accurate Biotechnology) on a LightCycler 480 II system (Roche, Switzerland). The *β*‐actin gene served as the internal control gene for data normalization. *Hmgb1*, *Tlr4*, *NF‐κB* p65, and *TNF‐α* mRNA expression were quantified via the 2^−ΔΔCT^ method. The corresponding primer sequences for these genes are detailed in Table 1.

**TABLE 1:** Primer sequences for qPCR.

Gene	Forward primer	Reverse primer
*β*‐actin	GGAGATTACTGCCCTGGCT CCTA	GACTCATCGTACTCCTG CTTGCTG
*Hmgb1*	TATGGCAAAAGCGGACAAG	CTTCGCAACATCACCAATG
*Tlr4*	TGAGCAGTCGTGCTGGTATC	GCCTTTCAGGTTCTTGGTGT
*NF‐κB* p65	AGGACCTGGAGCAAGCCATT CCAGGAGAAAGTCAGCCTCCT	TGGGTCAGAGGCCAAGTATG CCAGGAGAAAGTCAGCCTCCT

The primers were designed to ensure high specificity and efficiency for qPCR amplification, enabling accurate quantification of target gene expression levels.

### 2.13 Nissl Staining

At 28 days post‐injury, Nissl staining was performed on spinal cord sections encompassing the lesion epicenter. For each animal, surviving motor neurons in the ventral horn were quantified across three non‐consecutive sections by a blinded investigator using ImageJ. Neurons were counted only if they exhibited a visible nucleus/nucleolus, abundant Nissl substance, and a somatic diameter >30 µm; those with pyknosis or chromatolysis were excluded. Data are presented as the mean number of surviving motor neurons per section per animal.

### 2.14 Statistical Analysis

Statistical analyses were performed using GraphPad Prism 9.5.0. All data were first assessed for normality (Shapiro–Wilk test) and homogeneity of variances (Levene's test). Based on the experimental design, the choice of statistical test was as follows: Longitudinal behavioral data (BBB scores and inclined plate test) were analyzed using two‐way repeated measures ANOVA with treatment (Sham, SCI, EA, I, EA + I) and time (Day 1, 7, 14, 28) as the independent factors. Molecular and histological data (*n* = 5 per group per time point) obtained at terminal endpoints were analyzed by standard two‐way ANOVA. For all ANOVA models, when significant main or interaction effects were found, Tukey's post‐hoc test was applied for multiple comparisons. If the assumptions for parametric tests were violated, the non‐parametric Kruskal–Wallis test followed by Dunn's test was used. Pearson correlation coefficient (*r*) was calculated to assess the linear relationship between the protein expression levels of TNF‐α in the frontal cortex and the BBB scores at 14 and 28 days post‐injury. Data are presented as mean ± SEM, and a p < 0.05 was considered statistically significant.

## 3 Results

### 3.1 Electroacupuncture Improved Motor Function After SCI

**FIGURE 1:**
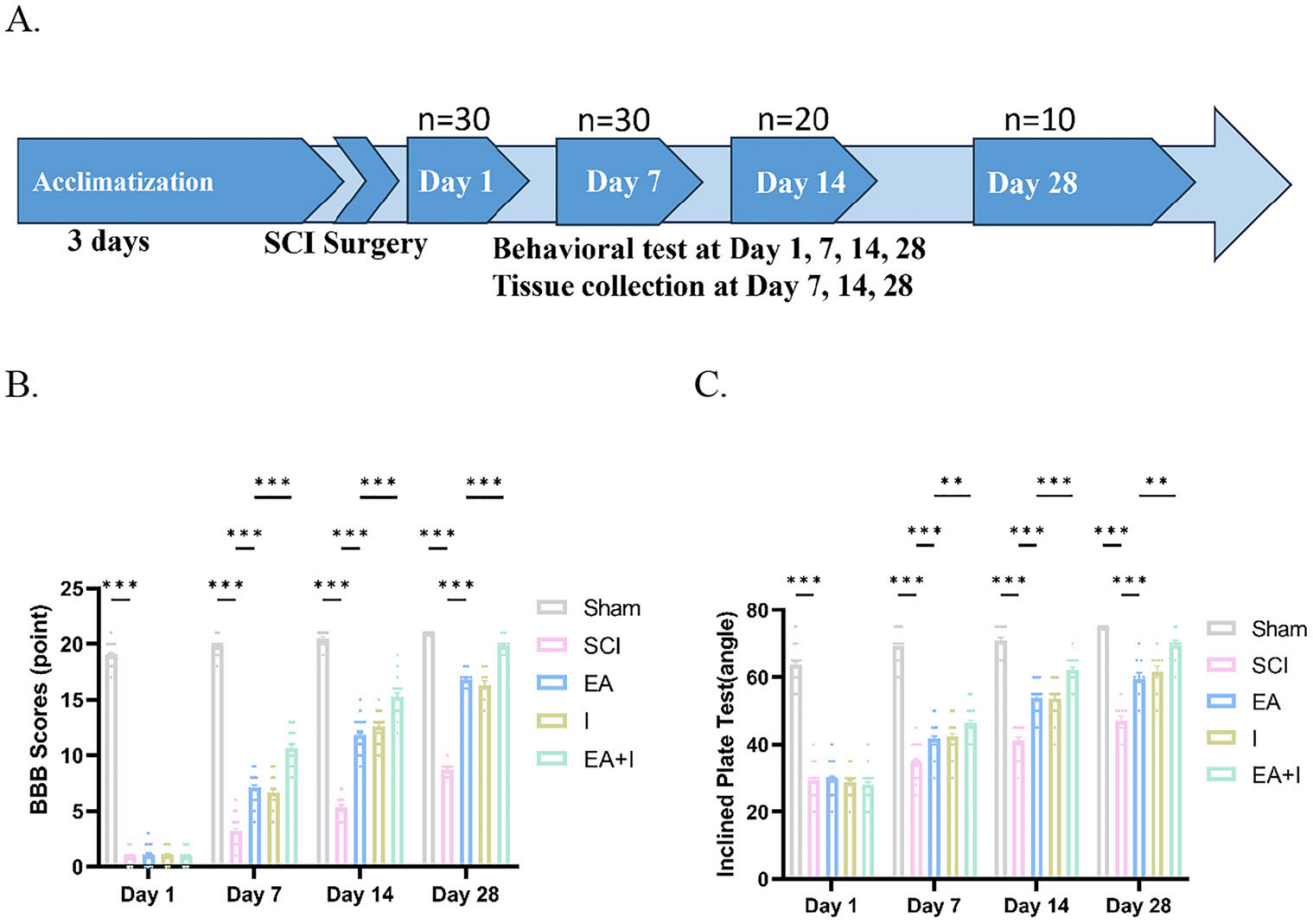
Timeline of the experiment and effects of EA on motor function in all groups of rats with SCI. (A) The timeline of the experiment, including acclimatization, the timepoint of behavioral assessment, and tissue collection (B) and (C). Assessment of hindlimb functional recovery using the (B) BBB scale and (C) inclined plate test. Data for both assessments are expressed as mean ± SEM. The sample size was n = 30 per group for the day 1 and 7 time points, n = 20 per group for the day 14 time point, and n = 10 for the day 28 time point. ***p < 0.001, **p < 0.01, and *p < 0.05 (two‐way ANOVA with Tukey's post‐hoc test).

Motor function was longitudinally assessed using the BBB locomotor rating scale and the inclined plate test at 1, 7, 14, and 28 days post‐SCI. As expected, all groups subjected to SCI exhibited severe motor deficits compared to the sham group at all tested time points (Figures 1B–C, *p* < 0.001).

On postoperative day 1, there were no significant differences in BBB scores or maximum holding angles among the SCI, EA, I, and EA + I groups, indicating a comparable initial injury severity across all experimental groups. However, beginning on day 7 and persisting through day 28, the therapeutic effects of the interventions became evident. Both EA and I conferred significant functional improvements, showing significantly elevated BBB scores and increased maximum holding angles compared to the SCI group (*p* < 0.001). The efficacy of these two groups was comparable, with no statistically significant differences observed between them.

Crucially, the EA + I group achieved significantly higher BBB scores and greater maximum holding angles compared to both the EA and I monotherapy groups starting from day 7 post‐injury and persisting through day 28 (*p* < 0.05 for all comparisons). This superior outcome is not merely indicative of a beneficial combination therapy. It provides a critical mechanistic proof. The fact that inhibition of HMGB1 can build upon and amplify the therapeutic gains of EA firmly established that the HMGB1 pathway is a primary mediator of EA's effects and a central driver of the post‐SCI recovery process.

### 3.2 Electroacupuncture Reduced Pro‐inflammatory Signaling via the HMGB1 Pathway in the Frontal Cortex After SCI

To evaluate the dynamic impact of EA on SCI‐induced supraspinal neuroinflammation, we analyzed the expression of HMGB1, TLR4, NF‐κB p65, and TNF‐α in the frontal cortex using both immunofluorescence and Western blot analysis at 7, 14, and 28 days post‐injury.

In the acute phase (Day 7), the SCI group exhibited a robust upregulation of all four pro‐inflammatory markers compared to the Sham controls (Figures 2A–H, *p* < 0.001). Intervention with either EA or the HMGB1 inhibitor (I) alone significantly attenuated these elevations. Notably, there was no statistically significant difference in protein expression between the EA and I groups. This parity suggests that EA treatment mimics the anti‐inflammatory efficacy of specific HMGB1 inhibition. However, the EA + I group yielded a more potent inhibitory effect, resulting in significantly lower expression levels of HMGB1, TLR4, NF‐κB p65, and TNF‐α compared to the EA group (*p* < 0.001).

FIGURE 2Effects of EA on reducing inflammation in the frontal cortex of rats following SCI. A‐D. Representative immunofluorescence images and quantification of HMGB1 (A), TLR4 (B), NF‐κB p65 (C), and TNF‐α (D) expression at 7, 14, and 28 days post‐SCI in five experimental groups: Sham‐operated control (Sham), SCI model (SCI), SCI treated with EA (EA), SCI treated with Inhibitor (I), and SCI treated with EA and inhibitor (EA + I). (Magnification: 400×, scale bar: 50 µm). E–H. Relative expression levels of HMGB1 (E), TLR4 (F), NF‐κB p65 (G), and TNF‐α (H) at 7, 14, and 28 days detected by WB. I–L. *Hmgb1* (I), *Tlr4* (J), *NF‐κB p65* (K), and *TNF‐α* (L) mRNA at both day 7 and day 28 timepoints detected by RT‐qPCR. Data are expressed as mean  ±  SEM. *n* = 5 per group per time point.***p < 0.001, **p <  0.01, and *p  <  0.05.

**Figure:**
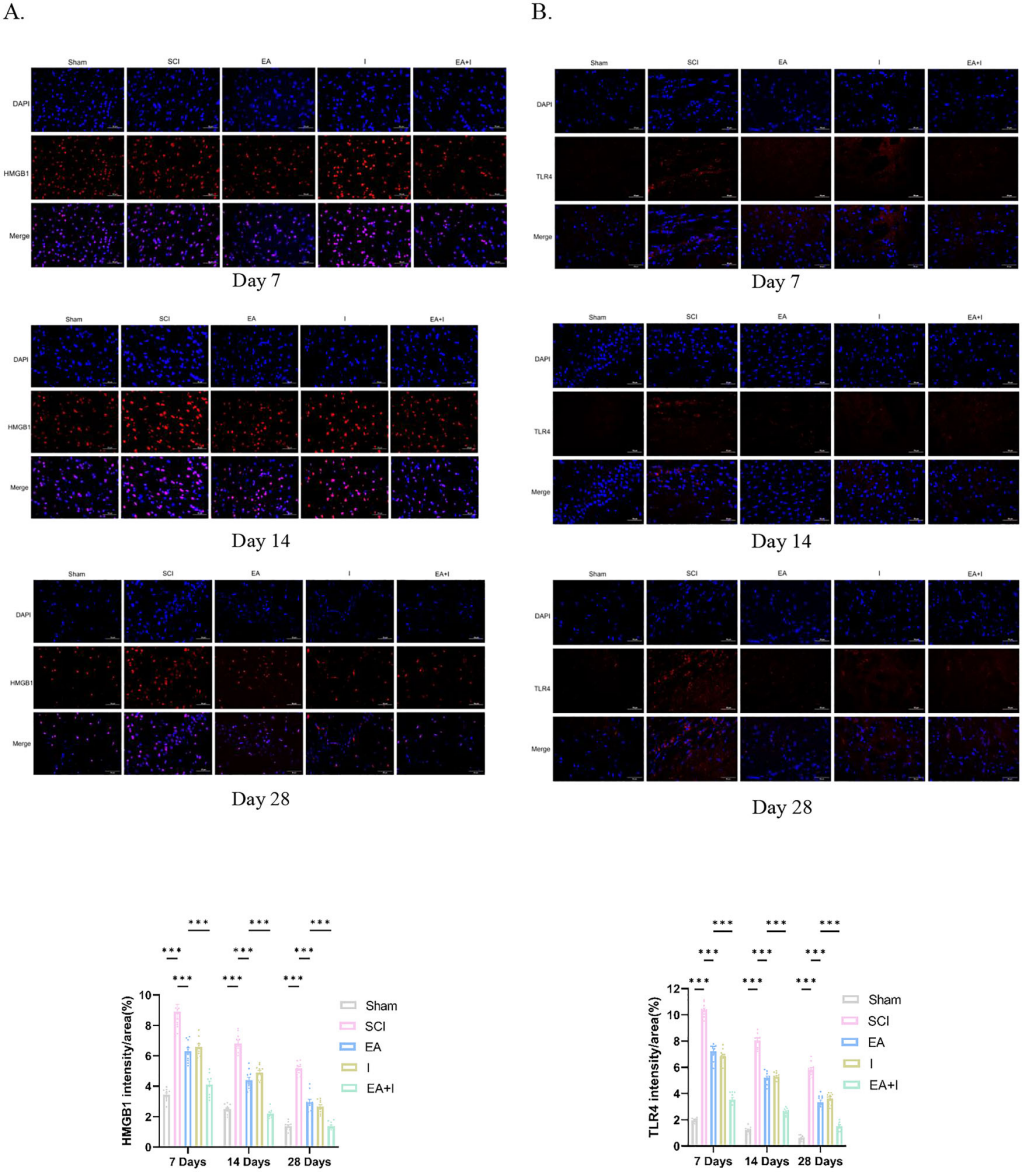

**Figure:**
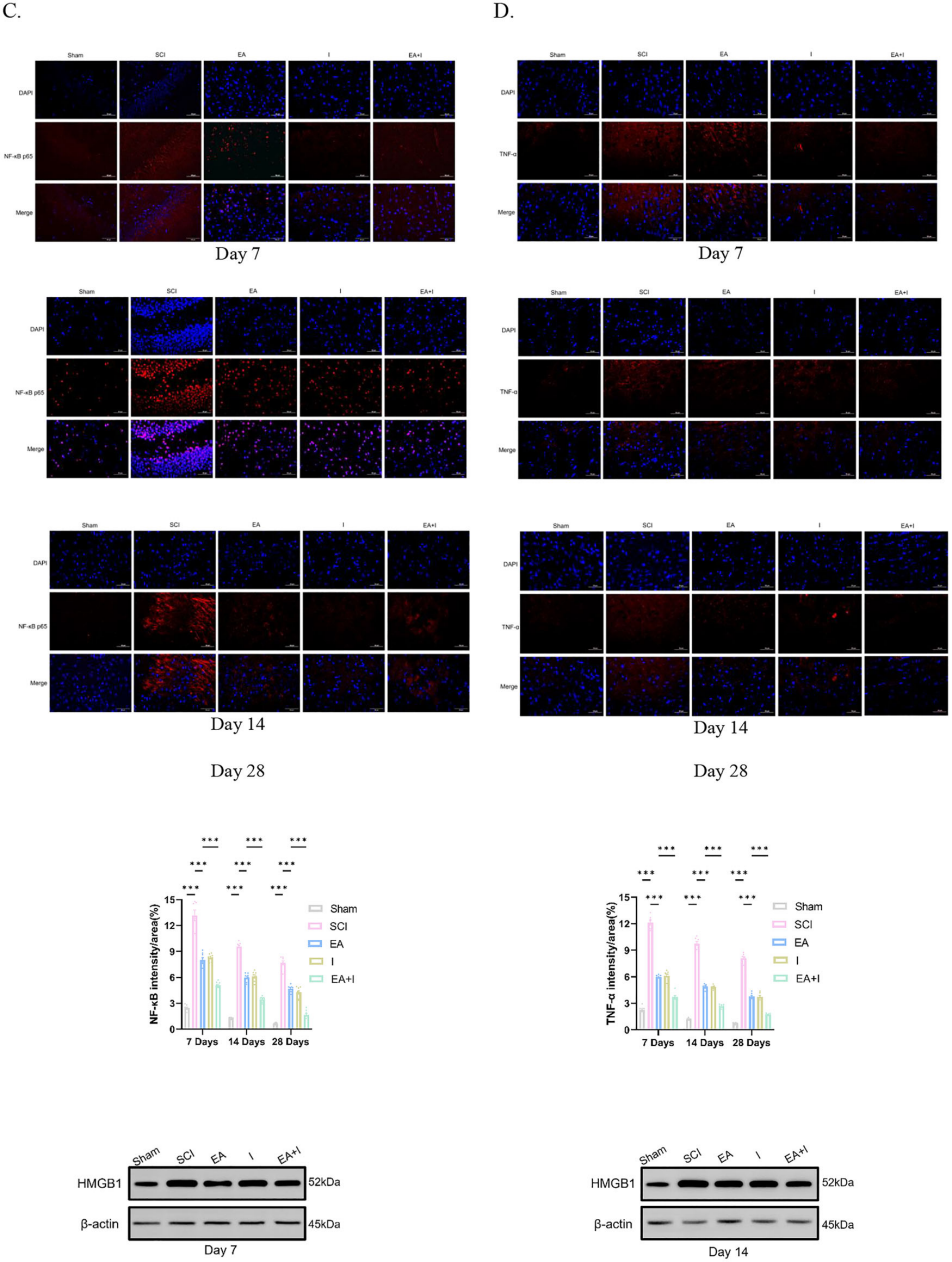

**Figure:**
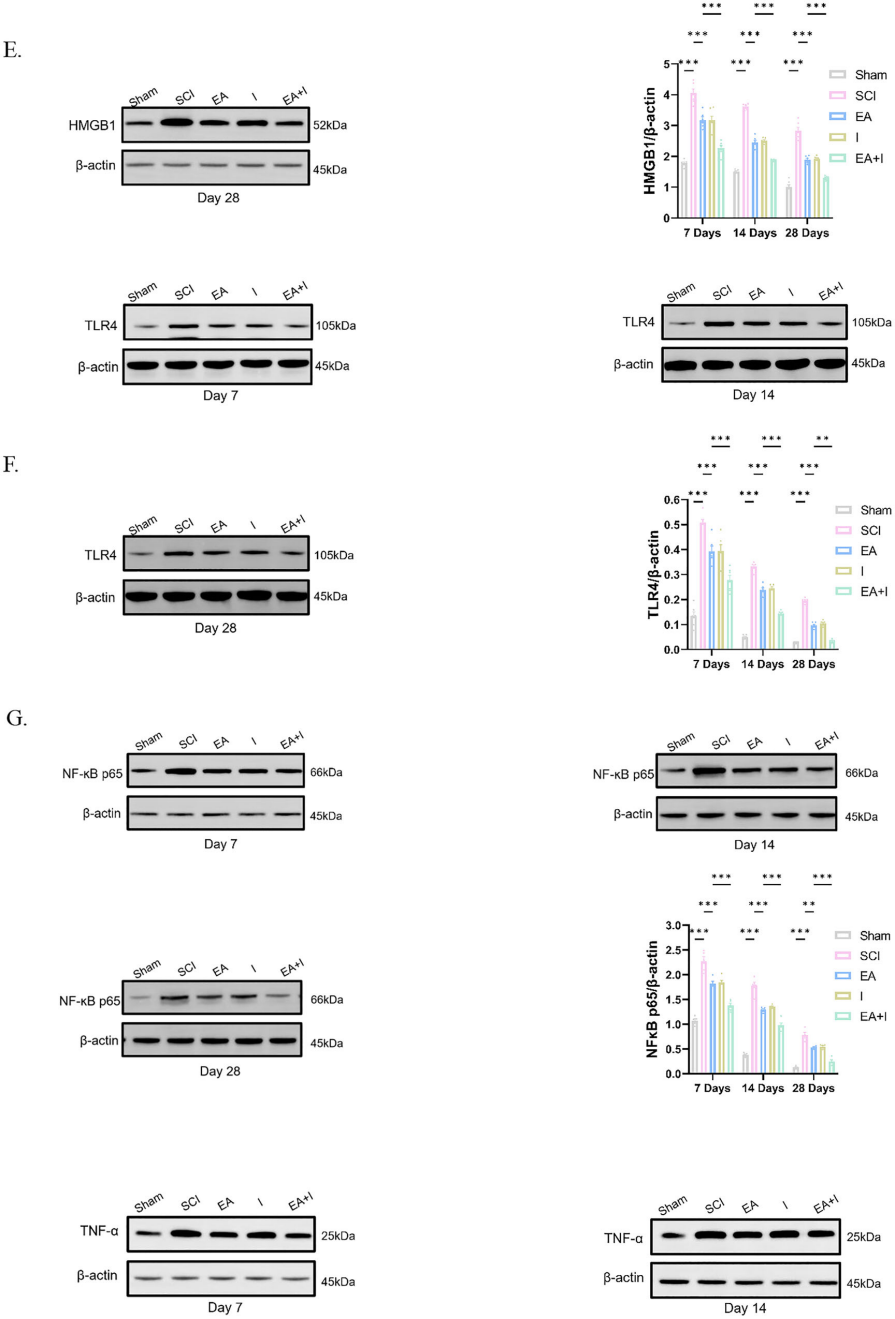

**Figure:**
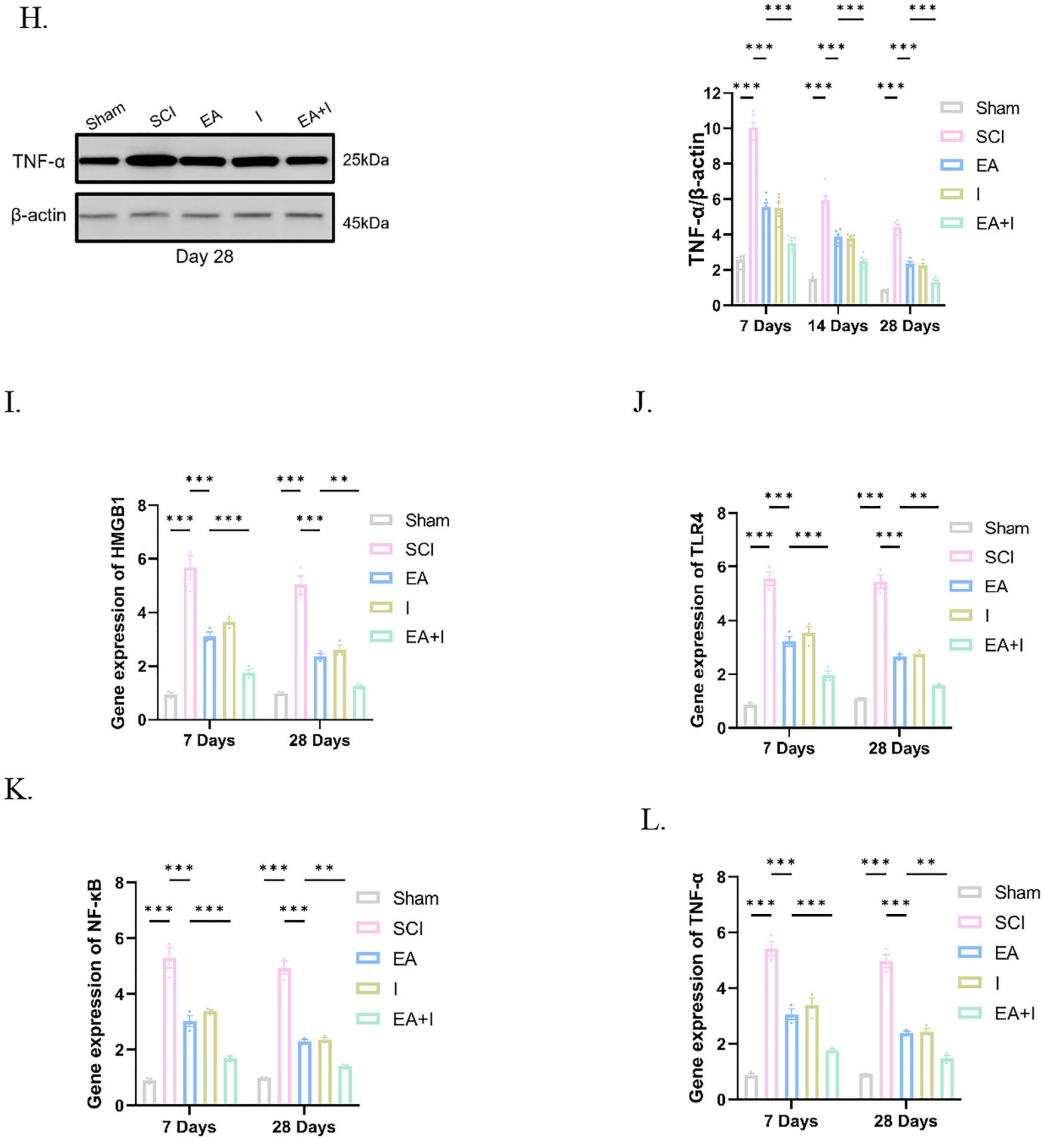

At 14 days post‐injury, this inflammatory trend persisted. While the absolute expression levels in the SCI group began to decline relative to the peak at Day 7, they remained significantly elevated compared to Sham controls (Figures 2A–H, *p* < 0.001). Western blot analysis confirmed that both EA and I groups continued to suppress the inflammatory cascade to a similar extent. Consistent with the acute phase, the EA+I group demonstrated superior efficacy, showing a further significant reduction in the expression of these pro‐inflammatory proteins compared to the EA group (Figures 2A–H, *p* < 0.001).

By Day 28 (chronic phase), although the expression of these markers in the SCI group had decreased further, a state of chronic neuroinflammation was evident as levels remained significantly higher than in Sham controls (Figures 2A–H, *p* < 0.001). Both EA and I treatments maintained their therapeutic effects; however, the EA+I group sustained the most profound suppression of neuroinflammation. At this terminal time point, the expression of HMGB1, TLR4, NF‐κB p65, and TNF‐α in the EA + I group remained significantly the lowest among all injury groups (Figures 2A–H, *p* < 0.001 vs. EA). Collectively, these findings confirm that EA exerts its neuroprotective effects by effectively blocking the HMGB1/TLR4/NF‐κB signaling axis, a mechanism that is further potentiated by pharmacological inhibition.

To investigate the transcriptional regulation of these mediators, we quantified their mRNA levels by RT‐qPCR. At 7 days post‐injury, the SCI group showed a dramatic increase in hmgb1, tlr4, NF‐κB, and TNF‐α gene expression (Figures 2I–2L, *p* < 0.001 vs. sham). EA treatment markedly attenuated these transcriptional increases. The suppressive effect was even more pronounced in the EA+I group, which exhibited significantly lower mRNA levels for all four targets compared to the EA group (*p* < 0.001). This pattern persisted at day 28, where gene expression in the SCI group remained elevated. Both EA and EA + I interventions continued to exert a significant suppressive effect, with the EA + I combination being the most effective at downregulating the expression of these pro‐inflammatory genes (Figures 2I–2L, *p* < 0.001 vs. EA).

### 3.3 Correlation Between Frontal Cortex Inflammation and Locomotor Recovery

**FIGURE 3:**
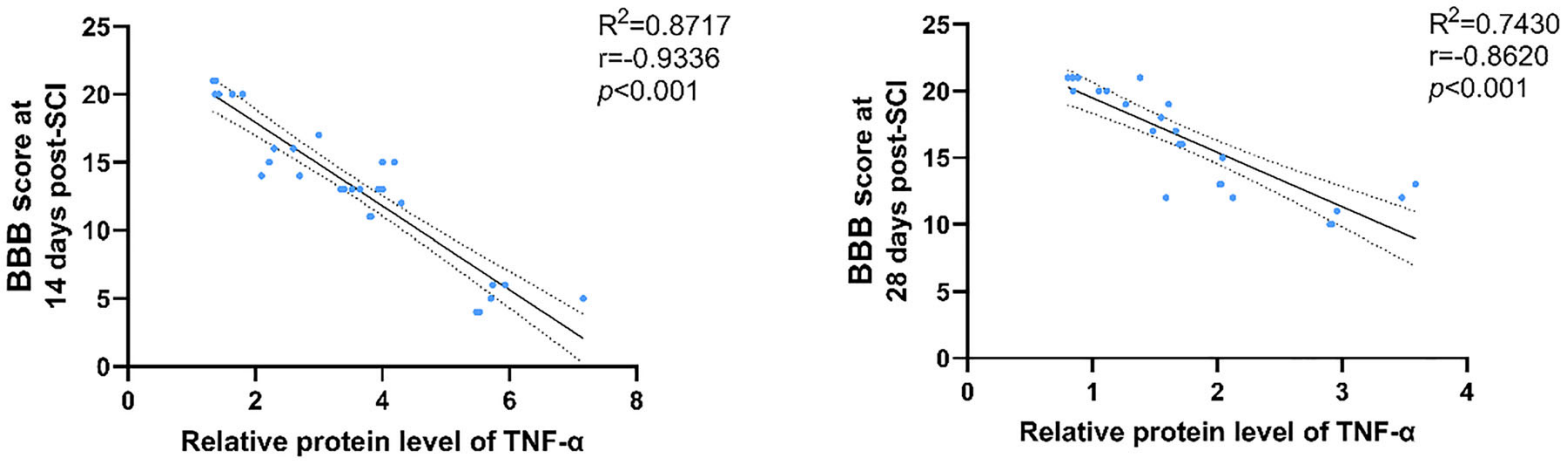
Frontal cortex TNF‐α expression is negatively correlated with locomotor recovery after SCI. Scatter plot showing the relationship between TNF‐α protein levels and BBB locomotor scores across all experimental groups at 14 and 28 days post‐injury. Statistical analysis (Pearson correlation) confirmed a significant negative correlation (Day 14: *r* = −0.9336; Day 28: *r* = −0.8620, *p* < 0.001 for both).

To investigate the potential relationship between supraspinal neuroinflammation and functional recovery, we performed a Pearson correlation analysis between TNF‐α protein expression in the frontal cortex and BBB scores at 14 and 28 days post‐injury. The analysis revealed a significant negative correlation (Figure 3; Day 14: *r* = −0.9336; Day 28: *r* = −0.8620, *p* < 0.001 for both), indicating that elevated inflammatory signaling in the frontal cortex is significantly associated with poorer locomotor outcomes following SCI.

### 3.4 Electroacupuncture Attenuated Microglial Activation in Frontal Cortex After SCI

**FIGURE 4:**
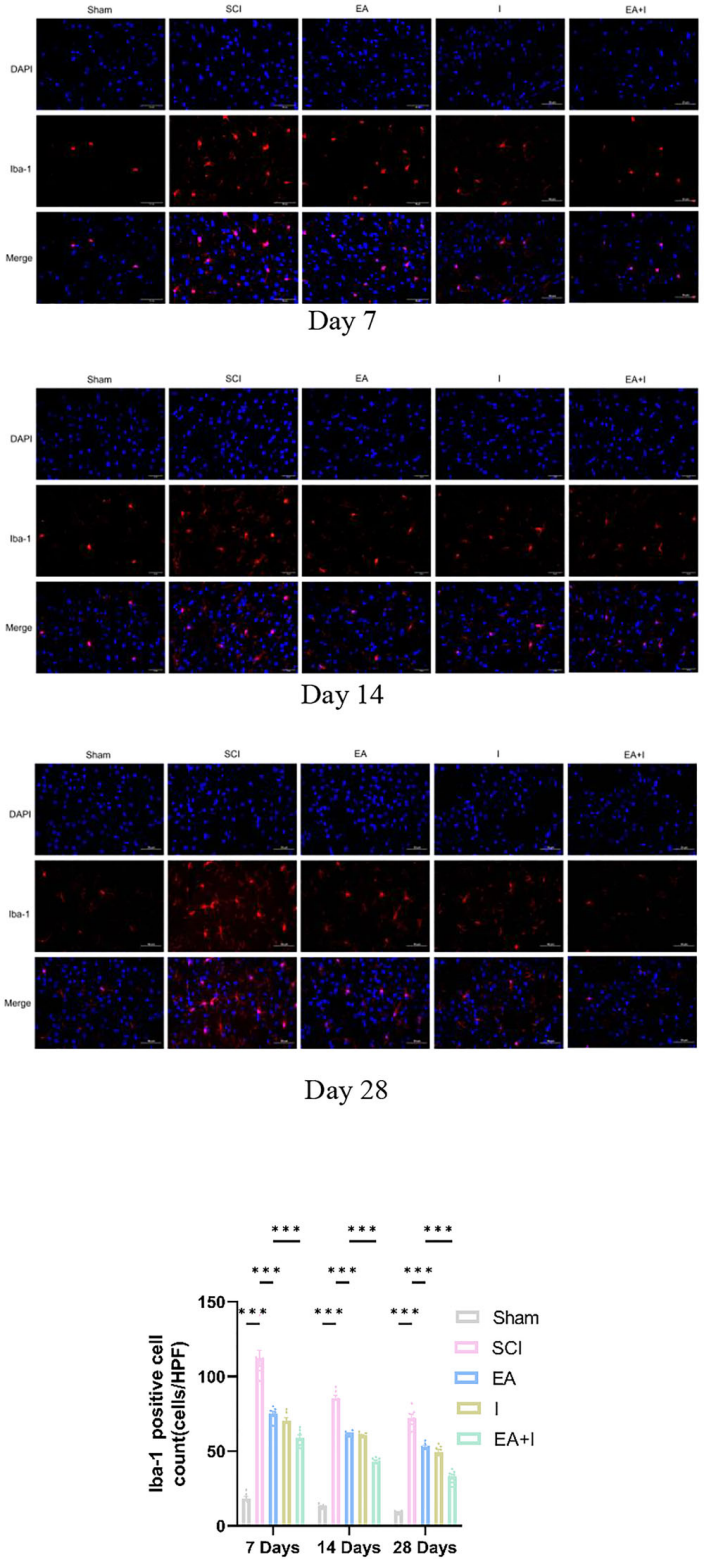
Effects of EA on microglial activation in the frontal cortex following SCI. Immunofluorescence images of Iba‐1 positive cells at 7, 14, and 28 days post‐SCI in five groups: Sham, SCI, EA, I, and EA + I. Lower panel shows the analysis of the Iba‐1 positive cell count at all timepoints (Magnification: 400×, scale bar: 50 µm). Data are expressed as mean  ±  SEM. *n* = 5 per group per time point. ***p <0.001.

To further investigate the anti‐neuroinflammatory effects of EA, we assessed microglial activation by quantifying Iba‐1 positive cells via immunofluorescence. Seven days post‐injury, the SCI group exhibited a marked proliferation of Iba‐1 positive microglia compared to the sham group (Figure 4, *p* < 0.001). EA intervention effectively counteracted this activation, leading to a substantial reduction in Iba‐1 positive cells versus the SCI group (*p* < 0.001). Notably, the EA+I group demonstrated a superior inhibitory effect, with significantly fewer Iba‐1 positive cells than the EA group (*p* < 0.05).

At 14 days post‐injury, characterizing the transition to chronic inflammation, the SCI group continued to exhibit a high density of activated, amoeboid microglia (Iba‐1 positive). Consistent with the molecular findings, EA treatment significantly reduced the number of Iba‐1 positive cells at this stage (*p* < 0.001 vs. SCI). The EA + I group showed further reduction compared to the EA group, indicating that continuous suppression of the HMGB1 axis remained more effective during this transitional phase.

By day 28, while microglial activation had partially subsided across all injury groups compared to day 7, the number of Iba‐1 positive cells in the SCI group remained significantly elevated above sham levels (*p* < 0.001). EA treatment continued to attenuate this chronic microglial activation (*p* < 0.001 vs. SCI). Crucially, the EA + I group maintained the lowest count of Iba‐1 positive cells, showing a significant difference compared to the EA group (*p* < 0.001).

### 3.5 Electroacupuncture Preserved Motor Neuron Survival in the Injured Spinal Cord

**FIGURE 5:**
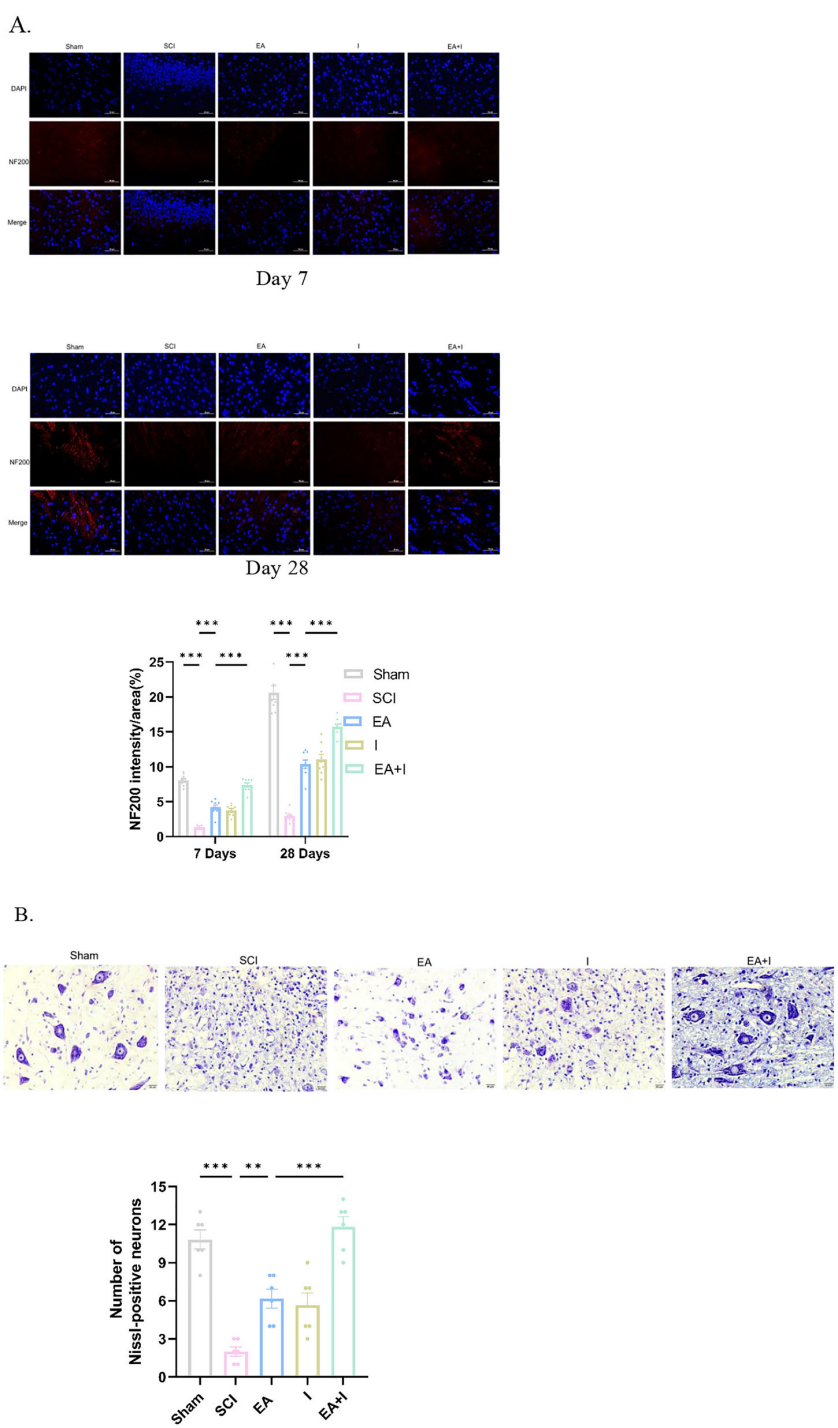
: EA promotes axonal and neuronal preservation in the injured spinal cord. (A) NF200 immunofluorescence (representative images and intensity quantification) in the lesional white matter at 7 and 28 days post‐SCI in five groups: Sham, SCI, EA, I, and EA + I (Magnification: 400×, scale bar: 50 µm) and (B) Nissl staining (representative images and motor neuron counts) in the ventral horn at 28 days post‐SCI in all five groups (Magnification: 400×, scale bar: 20 µm). Data are expressed as mean  ±  SEM. *n* = 5 per group. ***p < 0.001 and **p < 0.01.

Immunohistochemical analysis for NF200, a marker for large myelinated axons, was performed to evaluate axonal integrity. At both 7 and 28 days post‐injury, the SCI group exhibited a profound loss of NF200 immunoreactivity in the peri‐lesional white matter tracts compared to the sham group (Figure 5A, *p* < 0.001). In contrast, EA treatment significantly preserved NF200‐positive axons at both time points compared to the SCI group (*p* < 0.001). Notably, the EA + I group conferred superior neuroprotection, achieving a significantly higher density of intact NF200‐positive fibers than the EA group alone (*p* < 0.001).

To assess neuronal survival in the spinal cord ventral horn, Nissl staining was conducted at 28 days post‐injury. The SCI group demonstrated a dramatic loss of motor neurons, with significantly fewer surviving Nissl‐stained neurons compared to the sham group (Figure 5B, *p* < 0.001). Many remaining cells appeared shrunken and pale, indicating chromatolysis. EA intervention provided significant neuroprotection, as evidenced by a higher number of healthy, well‐stained neurons relative to the SCI group (*p* < 0.01). The most robust protective effect was observed in the EA + I group, which exhibited the highest count of surviving neurons among all injury groups and was significantly greater than that of the EA group (*p* < 0.001).

## 4 Discussion

This study demonstrates that EA confers multifaceted neuroprotective benefits following SCI in rats, improving motor function while suppressing the HMGB1/TLR4/NF‐κB neuroinflammatory axis in the remote frontal cortex at acute (day 7), subacute (day 14), and chronic (day 28) stages. We further established that this supraspinal anti‐inflammatory effect is mechanistically essential for EA's efficacy and is directly linked to the enhanced preservation of spinal motor neurons and their axonal integrity.

Notably, by mapping the temporal dynamics across acute, subacute, and chronic phases and demonstrating the spatial modulation from the spinal lesion to the supraspinal frontal cortex, our study provides a more comprehensive mechanistic understanding of EA's therapeutic actions.

### 4.1 EA Modulates the “Spinal Cord‐Brain” Inflammatory Axis via HMGB1/TLR4 Signaling

Our findings reveal that EA's therapeutic scope extends to the supraspinal level, specifically counteracting SCI‐induced neuroinflammation within the frontal cortex. This process is driven by damage‐associated molecular patterns (DAMPs), such as HMGB1, which propagates from the lesion site to activate microglia via TLR4 signaling (Chen et al. 2022; Sachdeva et al. 2018). Our results provide compelling evidence that EA directly counteracts this pathological inter‐regional signaling. We observed that EA suppressed the entire HMGB1/TLR4/NF‐κB cascade at both protein and mRNA levels.

Crucially, our data at 14 days post‐injury indicate that this pathological signaling is not merely an acute trigger but a sustained assault. The persistent elevation of HMGB1/TLR4/NF‐κB axis components in the SCI group suggests a critical temporal window where pro‐inflammatory signaling risks becoming chronic and maladaptive (Li et al. 2022). EA intervention during this window effectively truncated this continuous pathogenic input, not only dampening inflammation but potentially redirecting the cortical microenvironment towards a state more permissive for long‐term repair and plasticity (Zhao et al. 2024).

This finding is consistent with emerging evidence positioning EA as a potent modulator of this critical neuroinflammatory pathway (Wang et al. 2023; Wang et al. 2023). Furthermore, the significant negative correlation we observed between cortical TNF‐α levels and locomotor recovery (BBB scores) functionally links the attenuation of this supraspinal inflammatory cascade to improved motor outcomes. Notably, the correlation was even stronger at Day 14 (*r* = −0.9336) compared to Day 28 (*r* = −0.8620). This observation implies that the subacute transition represents a highly sensitive window where functional outcomes are closely tied to the inflammatory status (Bao and Yang 2023). It suggests that dampening neuroinflammation during this specific phase is a decisive factor in determining the long‐term recovery trajectory, thereby highlighting the critical importance of EA intervention during this time window.

### 4.2 The HMGB1/TLR4 Pathway Is a Critical Target for EA's Neuroprotective Effects

To investigate the mechanistic role of the HMGB1/TLR4 pathway, we introduced a specific HMGB1 inhibitor (Glycyrrhizin). The finding that its co‐administration with EA (EA + I group) produced an additive effect provides crucial functional evidence that the HMGB1/TLR4/NF‐κB axis is a critical target for EA's neuroprotection. The observation that combining EA with an HMGB1 inhibitor yields superior outcomes suggests that while EA significantly downregulates this pathway, it may not completely abolish the massive release of HMGB1 induced by the primary trauma. Therefore, the pharmacological inhibitor acts synergistically to neutralize residual HMGB1, maximizing the anti‐inflammatory benefit. The fact that pharmacological inhibition of HMGB1 neither occluded nor replaced EA's efficacy but rather augmented it suggests that EA's action on this pathway is significant, though may not be exhaustive. This result strongly positions the suppression of the HMGB1/TLR4/NF‐κB axis as a primary, though potentially not exclusive, mechanism through which EA exerts its benefits. This additive effect confirms that targeting this axis is the correct strategy, and EA is a potent physiological modulator of this target.

### 4.3 Supraspinal Neuroprotection Translates to Preservation of Spinal Motor Neurons and Axons

A crucial question is how attenuating neuroinflammation in the frontal cortex translates to structural preservation at the distant spinal lesion site. We propose a well‐supported hypothesis centered on EA's neuroprotective effect on “upstream” corticospinal neurons. Following SCI, the transection of descending motor pathways induces significant retrograde degeneration in the parent neuronal cell bodies within the motor cortex (Tsuboguchi et al. 2023; Yang and Martin 2023; Zareen et al. 2017). This process is exacerbated by local neuroinflammation, which can trigger apoptosis of these crucial upper motor neurons (Henry and Loane 2025; Li, Ritzel, et al. 2020a).

Our study provides direct structural evidence supporting this hypothesis. For the first time, using Nissl staining, we demonstrated that SCI caused a profound loss of surviving motor neurons in the ventral horn. EA treatment, however, significantly preserved the number of these viable neurons, which are characterized by a clear nucleus and abundant Nissl substance. This preservation of “upstream” neuronal bodies is the likely reason for the enhanced integrity of their “downstream” axons, as evidenced by the robust NF200 immunoreactivity at the lesion site. Critically, the neuroprotective effect of EA on neuron survival was further cemented by the HMGB1 inhibitor findings, bridging the gap between supraspinal anti‐inflammation and spinal structural integrity. This repositions EA as a holistic therapy that protects the entire corticospinal motor unit, with supraspinal neuroprotection emerging as a critical mechanism.

### 4.4 EA Promotes Axonal Preservation and Mitigates Microglial Activation

Our findings demonstrate that EA's benefits culminate in tangible structural improvements. The significant reduction in Iba‐1 positive cells in the frontal cortex indicates that EA effectively curbs pathological microgliosis, a hallmark of chronic neuroinflammation. This aligns with studies showing EA's ability to regulate microglial activation and polarization (Pei et al. 2022; Zhao et al. 2017). Concurrently, the robust preservation of NF200 immunoreactivity at the lesion site underscores EA's capacity to support axonal integrity. This structural preservation is the likely anatomical correlate of the observed functional recovery and may result from a combination of the direct “upstream” neuroprotection discussed above and other local anti‐inflammatory and pro‐regenerative effects of EA (Wang et al. 2024; Xiao et al. 2022).

### 4.5 Limitations and Future Directions

While our findings establish clear and mechanistically supported links between EA, inhibition of the HMGB1/TLR4 axis, and neuroprotection, certain limitations should be acknowledged to guide future research.

#### 4.5.1 Genetic Validation

In this study, we employed pharmacological inhibitors to verify the necessity of the HMGB1/TLR4 pathway. While these results are robust, future studies employing TLR4 or HMGB1 knockout animal models would provide gold‐standard genetic evidence to further corroborate our findings.

#### 4.5.2 Comprehensive Neuroglial Response

Our analysis primarily focused on microglia/macrophages. However, astrocytes are also key players in neuroinflammation and glial scar formation. Future investigations should include markers like GFAP to explore EA's impact on the astrocytic response and its interaction with microglia.

#### 4.5.3 Methodological Refinements

Protein quantification: We acknowledge that quantifying the p‐p65/total p65 ratio is the standard for assessing NF‐κB activation. Although sample exhaustion of the frontal cortex tissues prevented this specific analysis in the current study, the consistent downregulation of upstream TLR4/HMGB1 and the downstream effector TNF‐α provides strong evidence of pathway inhibition. Future work will prioritize this ratio for more rigorous normalization.

Secreted factors: To better capture secreted protein dynamics in the extracellular space, future work could incorporate ELISA for cytokine measurement alongside Western blotting.

Temporal dynamics of molecular, transcriptional, and structural changes: While our protein data delineate a continuous suppression of the HMGB1/TLR4 axis from acute to chronic stages, our complementary qPCR analysis was conducted only at the Day 7 and Day 28 endpoints. Similarly, our structural analyses of the spinal cord tissue were focused on these two timepoints. Future studies incorporating qPCR at the subacute transition (e.g., Day 14) alongside NF200 and Nissl staining will be essential to precisely correlate the full temporal profile of supraspinal anti‐inflammatory signaling with the dynamic morphological preservation of the corticospinal tract.

## 5 Conclusion

In conclusion, our study provides compelling evidence that EA promotes significant functional and structural recovery after SCI. We demonstrate that a key mechanism underlying these benefits is the suppression of the “spinal cord‐brain” neuroinflammatory axis. Specifically, EA inhibits the HMGB1/TLR4/NF‐κB signaling pathway in the frontal cortex. The functional necessity of this mechanism was decisively shown through our inhibitor experiment, where specific blockade of HMGB1 significantly amplified EA's therapeutic effects. This result confirms the HMGB1 pathway not only as a mediator but also as a pivotal and specific target for EA's restorative action. This sustained supraspinal modulation restrains microglial overactivation, leads to the direct preservation of surviving motor neuron cell bodies and their axons in the spinal cord, and ultimately culminates in improved locomotor function. These findings highlight EA as a potent, multi‐target therapy that orchestrates a favorable microenvironment for neural repair by targeting a critical upstream inflammatory cascade.

## Author Contributions

**Yu Ning**: conceptualization, methodology, data curation, software, and writing – original draft. **Xin Hao**: investigation, writing – review and editing. **Yifei Dong**: formal analysis, writing – review and editing. **Rattanasakon Phattharapon**: validation. **Keduo Liu**: investigation. **Yuting Lin**: methodology. **Ying Yang**: data curation. **Yuping Mo**: supervision, project administration, and funding acquisition. **Suhua Shi**: supervision, resources, writing – review and editing. **Zhigang Li**: supervision, project administration, funding acquisition, writing – review and editing. Yu Ning, Xin Hao, and Yifei Dong contributed equally to this work as co‐first authors. All authors have reviewed and approved the final version of the manuscript.

## Funding

This research was supported by the National Natural Science Foundation of China (Nos. 82374593 and 82305381) and Shenzhen High‐level Hospital Construction Fund (No. 24275G1001).

## Ethics Statement

All experimental procedures complied with the guidelines of the Principles of Laboratory Animal Care formulated by the National Institutes of Health and the legislation of the People's Republic of China for the use and care of laboratory animals. The experimental protocols were authorized by the Animal Experimental Center of Beijing University of Chinese Medicine (BUCM2024051002‐2081).

## Conflicts of Interest

The authors declare no conflicts of interest.

## Data Availability

The raw data supporting the conclusions of this article will be made available by the authors, without undue reservation. The data that support the findings of this study are available in zenodo.org at https://doi.org/10.5281/zenodo.18153454.
